# Drug response profile-based machine learning enables strategic cell line and compound selection for drug development

**DOI:** 10.1093/bioinformatics/btag293

**Published:** 2026-05-08

**Authors:** Abbi Abdel-Rehim, Emma Tate, Larisa N Soldatova, Ross D King

**Affiliations:** Department of Chemical Engineering and Biotechnology, University of Cambridge, Cambridge, CB3 0AS, United Kingdom; Arctoris Ltd, Abingdon, OX14 4SA, United Kingdom; Department of Mathematics, University College London, London, WC1H 0AY, United Kingdom; Department of Chemical Engineering and Biotechnology, University of Cambridge, Cambridge, CB3 0AS, United Kingdom; Department of Biology and Biological Engineering, Chalmers University of Technology, Gothenburg, 412 96, Sweden; Department of Computer Science and Engineering, Chalmers University of Technology, Gothenburg, 412 96, Sweden; The Alan Turing Institute, London, NW1 2DB, United Kingdom

## Abstract

**Motivation:**

Early-stage drug discovery relies on testing compounds across a limited set of cell lines, making it challenging to capture biological diversity while maintaining experimental efficiency. Current predictive approaches for identifying responsive cell lines often depend on high-dimensional omics data, which can be costly and difficult to interpret. We therefore evaluated whether drug-response panel (DRP) descriptors, which capture sensitivity profiles to a reference set of compounds, can provide an efficient and informative alternative for modelling drug response in cell lines.

**Results:**

Using gradient boosting models across GDSC and CCLE datasets, DRP descriptors consistently outperformed mRNA expression features in predicting drug sensitivity (−log10(IC_50_)), although performance varied across compounds. DRP-guided cell line selection enabled downstream omics-based modelling that recovered known MAPK-associated sensitivity signatures and identified potential biomarkers for MEK1/2 and BTK/MNK inhibitors. Extending this framework, we demonstrated its utility in compound prioritisation by distinguishing between tumourigenic MCF7 and non-tumourigenic MCF10A cells, successfully identifying compounds with selective activity. Together, these results show that DRP-based representations, derived from compact screening panels, support efficient cell line selection, biomarker discovery, and compound prioritisation in early-stage drug development.

**Availability:**

Code and data uploaded to https://github.com/abbiAR/-Strategic-Cell-Line-and-Compound-Selection-Using-Drug-Response-Profiles

## 1 Introduction

The development of cancer therapies often begins by identifying a specific molecular target, followed by the optimisation of a drug to achieve strong affinity and specificity. Initial efficacy is typically evaluated in vitro using cell culture models. However, the inherent heterogeneity of cancer necessitates testing across multiple cell lines to confirm the compound’s intended toxicity. At this early stage, testing is usually limited to pre-selected cell lines based on target validation and tissue type. Given the extensive diversity of available cell lines, and the resource-intensive nature of these experiments, it is impractical to evaluate every candidate compound across a comprehensive range of cell line models. Selecting a small, informative subset of cell lines that capture biological diversity while minimising experimental effort can accelerate discovery and improve later-stage success.

Cell line assays provide crucial insights into a drug’s mode of action, which is particularly important for targeted therapies (Goodspeed *et al*. 2016, Gonçalves *et al*. 2020). These insights can also reveal biomarkers indicative of drug response, which enhances the probability of success during the challenging transition from in vitro models to in vivo studies and clinical trials (Barretina *et al*. 2012, Li *et al*. 2021). While tissue of origin shapes the genomic landscape, it often proves too coarse to effectively guide targeted therapies. For any tissue type, drugs appear to be effective only in subpopulations (Adashek *et al*. 2024), even where a genetic marker has been identified (Iorio *et al*. 2016, Jordan *et al*. 2017, Gouda *et al*. 2023, Wahida *et al*. 2023). Thus, early and efficient identification of molecular determinants of response is critical. A key strategy is to predict and select an optimised set of cell lines highly sensitive to a compound, which can then be used in focused mechanistic studies to extract relevant biological information and robust biomarkers.

**Figure 1:**
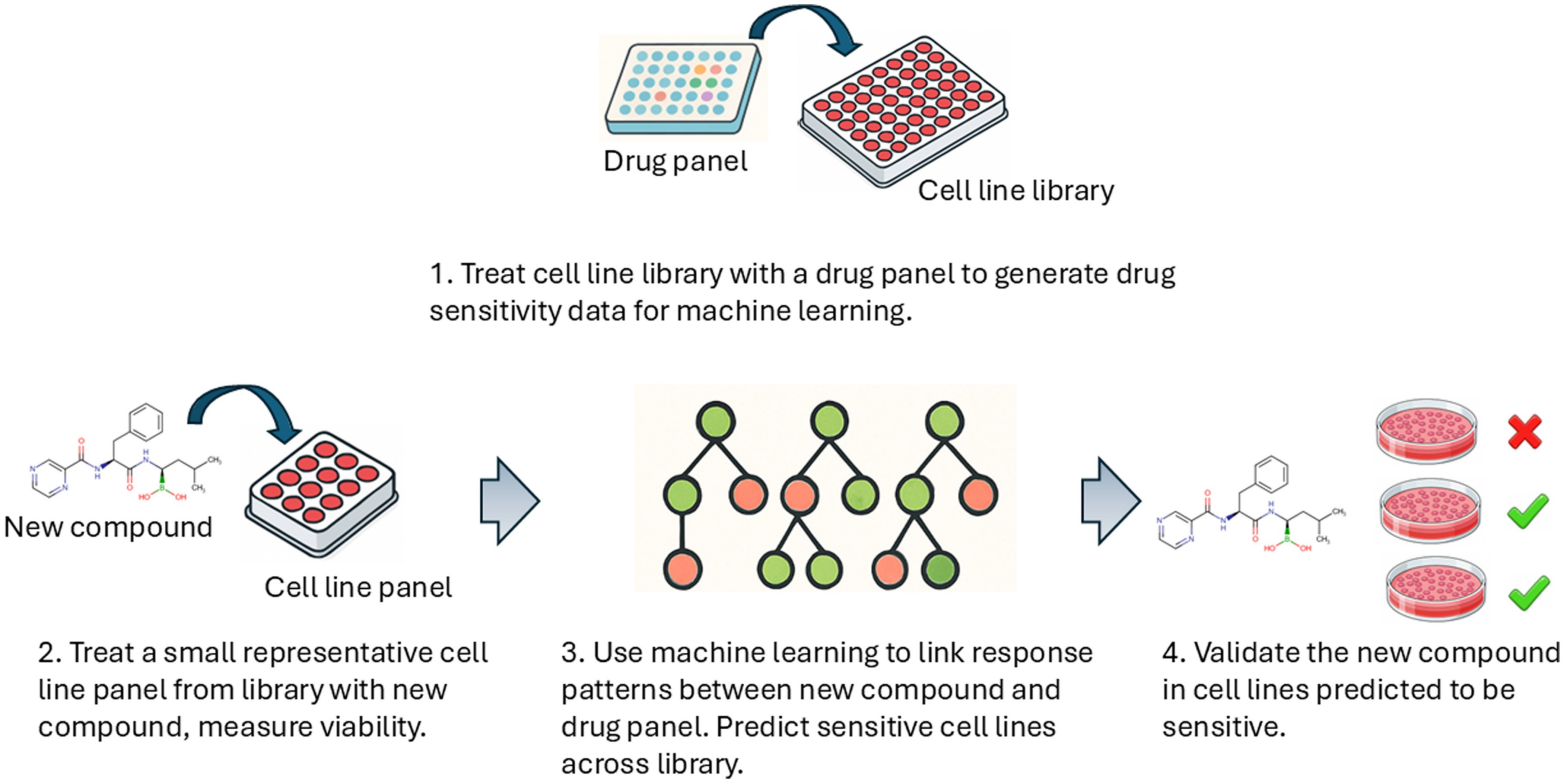
Concept of DRP-based prediction for new compound activity. Cell lines are characterised by their response to a fixed drug panel. New compounds are tested in a subset of these cell lines. Machine learning is used to learn relationships between the new drug’s responses and those of the drugs in the panel. The generated model is then used to predict the activity of the new drug in other cell lines that have been screened with the drug panel but not yet tested with the new drug.

Recent efforts have turned to omics profiling to guide drug sensitivity predictions; however, these approaches often require large sample sizes, suffer from limited generalisability across drugs, and can be challenging to interpret (Menden *et al*. 2019, Xia *et al*. 2024, Baião *et al*. 2025). Emerging descriptor-based models, such as drug response panels (DRPs), offer a promising alternative by capturing phenotypic drug sensitivity profiles without relying solely on molecular data (Moshkov *et al*. 2023, Dolgin *et al.* 2024, Abdel-Rehim *et al*. 2025). In this framework, each cell line is represented by a vector of drug response measurements across a fixed reference panel of compounds. These response vectors serve as functional descriptors of the cellular state, implicitly encoding pathway activity and compensatory mechanisms. Yet, their comparative advantages and integration into early-stage drug discovery workflows remain underexplored. In this study, we develop predictive models for drug activity in cell lines, systematically comparing omics-based features to DRP descriptors using gradient boosting decision tree models (Fig. 1).

**Figure 2:**
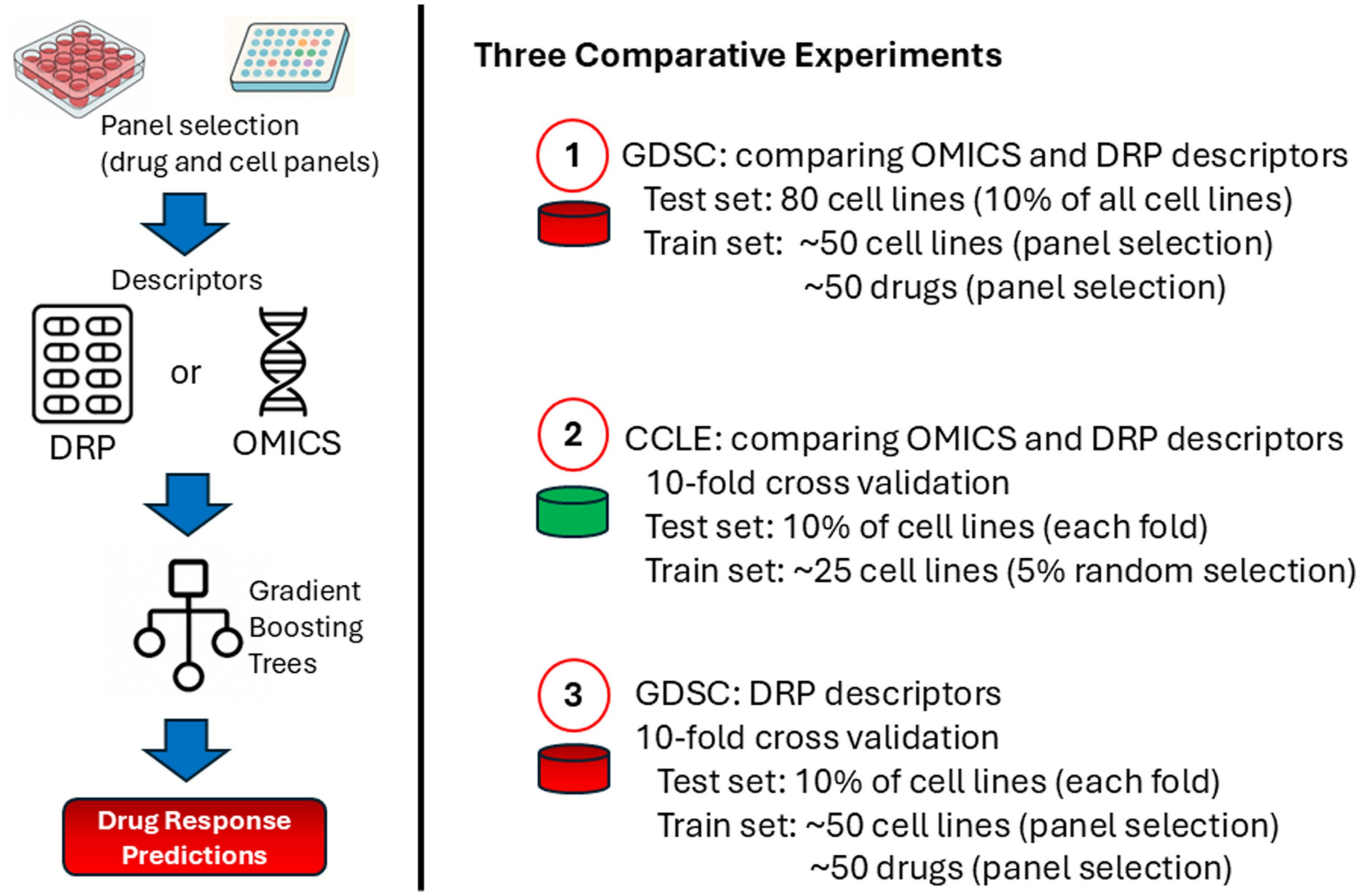
Three benchmarking experiments. (1) Comparing performance of the omics and DRP descriptors on the GDSC dataset using a dedicated test set. (2) Comparing performance of the descriptors on the CCLE dataset using cross validation. (3) Comparing performance of DRP descriptors when considering all datapoints in the GDSC dataset or each drug individually.

We show that DRP descriptors outperform traditional omics in predicting drug sensitivity using Genomics of Drug Sensitivity in Cancer (GDSC) and Cancer Cell Line Encyclopedia (CCLE) datasets. We also examined DRP descriptor performance on the GDSC dataset in more detail, comparing evaluation across all drug-cell line pairs versus on a per-drug basis (Fig. 2). Using complementary explainability approaches, including feature-response correlations, random forest feature importance, and SHAP (SHapley Additive exPlanations) analysis, we confirmed known MAPK-inhibitor sensitivity signatures and uncovered novel potential candidates, including regulators of Rho GTPases, Ras/ERK and BCL-2 pathways. These findings highlight the ability of even moderately sized cell line panels to yield biologically meaningful insights.

Lastly, we tested whether DRP-based machine learning models could guide prospective compound selection to distinguish between cancerous and non-cancerous epithelial lines. Focusing on breast epithelial MCF7 versus non-tumourigenic MCF10A cells, our models successfully nominated compounds with divergent, lineage-specific effects, validated experimentally. Overall, this work demonstrates that predictive modelling based on DRP descriptors offers a robust and efficient strategy for early-stage drug discovery. By enabling rational cell line selection, uncovering biologically meaningful response determinants through explainability analyses, and prospectively nominating compounds that discriminate between cancerous and non-cancerous breast epithelial cell types, our approach provides a scalable framework to enhance compound prioritisation and guide biomarker discovery early in the drug development pipeline.

## 2 Methods

Datasets: Drug response data were obtained from Genomics of Drug Sensitivity in Cancer (GDSC) datasets 1 and 2 (IC_50_ values—https://www.cancerrxgene.org/), CCLE (IC_50_ values—https://depmap.org/), and ChEMBL (v.35): https://www.ebi.ac.uk/chembl/, as well as NCI60 (March 2025): https://dctd.cancer.gov/data-tools-biospecimens/data. mRNA expression profiles were obtained from Sanger (TPM values—https://cellmodelpassports.sanger.ac.uk/).

All datasets were processed and consolidated. The GDSC datasets contained overlapping drugs, and some drug and cell names varied between datasets. We manually aligned the cell lines with differing names and compared drugs using their MACCS fingerprints to remove redundant entries. Non-small molecule drugs and those without a structure for MACCS fingerprinting were excluded. Drugs with over 90% cell line coverage, and cell lines tested against at least 90% of these drugs were retained. This resulted in a unified GDSC dataset of 806 cell lines and 293 drugs. The CCLE dataset contained 24 drugs measured against ∼500 cell lines. The mRNA data was log2-transformed (log2(TPM + 1)), followed by dimensionality reduction using scikit-learn’s (v.1.2.0) implementation of PCA, retaining components that accounted for 90% of the variance in the dataset (388 features). PCA was not applied for the explainability studies.

To promote reproducibility and benchmarking, we have uploaded the processed GDSC and CCLE datasets containing −log10(IC_50_) values, along with clearly labelled cell and drug names. The mRNA expression datasets containing log2(TPM + 1) values (including 7999 genes exceeding a variance of 1 across 806 cell lines), along with PCA-transformed data used in model building, are also available.

MACCS fingerprints: MACCS molecular fingerprints were generated using RDKit (v.2022.09.3), from PubChem SMILES corresponding to the drug names in the datasets, for all molecules with available SMILES.

Panel Selection: Drug response panels (DRPs) were defined as vectors of −log10(IC_50_) values corresponding to a selected reference set of drugs. If the panel contained *k* compounds, each cell line was represented by a k-dimensional vector describing its sensitivity profile, serving as a functional descriptor of cellular state (Abdel-Rehim *et al*. 2025).

Cell line panels were defined as reduced subsets of cell lines designed to capture the majority of variance in the drug response matrix, enabling construction of minimal yet information-rich screening panels.

DRPs and cell line panels were selected using the same procedure. Samples (drugs or cell lines) in the bottom 50% of response variance were excluded. A Pearson correlation matrix was computed among the remaining samples, which were ranked by median correlation to all others. Selection began with the sample exhibiting the lowest median correlation to all other samples. Once selected, all remaining samples with a Pearson correlation above a user-defined threshold to this sample were removed from consideration. The procedure was then repeated with the next lowest-correlated remaining sample and continued iteratively until no eligible samples remained. The correlation threshold was adjustable to control panel stringency. For each experiment, a random subset of 70% of training set cell lines was used for panel selection to prevent identical selections across experiments. Panel construction was performed using training data only. Panel selection typically resulted in a set of 50 drugs and 50 cell lines used for model construction.

Machine Learning: We employed the scikit-learn implementation of Gradient Boosting Trees (v.1.2.0) for drug activity prediction (Pedregosa *et al*. 2011). Cancer cell lines were randomly allocated into training (80%), validation (10%), and test (10%) sets. All panel selection and model fitting were performed using training data only. Models were trained separately for each drug, but evaluation was performed across all drug-cell line pairs in the test set, unless explicitly stated otherwise. Target values were bioactivity measurements from cell line viability assays −log10(IC_50_). Initial experiments using mutation and CNV descriptors alongside mRNA profiles did not yield notable performance improvements and were therefore not included.

Hyperparameter Optimisation (GDSC): To optimise model complexity, 58 drugs (approximately 20% of the total) were randomly selected from the GDSC dataset. Gradient boosting trees were evaluated using tree depths of 2–4 and 50 or 100 trees across five independent experiments. For each experiment, drug panel selection was performed using the training set, yielding panels of approximately 40–50 drugs. Models were trained using a randomly selected subset of 250 cell lines from the training data, consistent with previous observations that increasing training size beyond this range provides limited additional performance gains (Abdel-Rehim *et al*. 2025). As no significant differences were observed across configurations (Table S1, available as supplementary data at *Bioinformatics* online), the simplest model (50 trees, depth 2) was selected and used for all subsequent GDSC analyses. The same configuration was subsequently applied to the CCLE dataset without additional hyperparameter optimisation yielding consistency across datasets.

Explainability: For the explainability analysis, we focused on three drugs: Refametinib (MEK1/2 inhibitor), PD0325901 (MEK1/2 inhibitor), and QL-X-138 (BTK/MNK inhibitor).

Cell lines were ranked based on DRP-predicted sensitivity. A refined panel was constructed consisting of the initially selected lines from the cell line panel plus the top and bottom 10 predicted responders. For each experiment (*n* = 3) and drug, tree-based models were built using the RandomForestRegressor (200 trees, default settings, scikit-learn v.1.2.0) to facilitate stable feature importance estimation and SHAP-based interpretation. The 500 genes with the highest absolute correlation to the drug sensitivity across the selected cell lines were used as descriptors, providing a dimensionality reduction step within the refined cohort while retaining relevant response signals. Feature importances were extracted using the built-in feature_importances_() function. SHAP analysis was performed using the Python shap package, with SHAP values calculated on held-out data not used for model training to prevent information leakage and ensure independent interpretation.

Predicting drug responses for experimental validation: To predict responses in MCF10A and MCF7, the drug response data for MCF10A, serving as the DRP, was sourced from ChEMBL. Many of these drugs had been measured in few or no other cell lines. To address this, QSAR models were built based on cell lines in the NCI60 dataset (top 54 cell lines based on compound coverage spanning thousands of compounds), using MACCS fingerprints to predict the activity (−log10(IC_50_) values) of the 223 compounds that had been measured in MCF10A (featured in ChEMBL). Given the substantially larger training set used for QSAR modelling, model capacity was increased (100 trees, maximum depth 4) to better capture structure-activity relationships.

Resultant predictions for the 223 compounds were used to establish a unified DRP across all cell lines and train a DRP-based predictive model. Except for MCF10A, all lines are included in the GDSC drug library (which covers the drug library used for experimental validation). GDSC data hence served as the known values to train the model and predict activities for both MCF10A and MCF7 across a library of 157 drugs. Although MCF7 had experimental measurements for many compounds, we used predicted activities to showcase the method’s predictive performance for both cell lines.

Drug Screening: The drug library consisted of 157 compounds, see Table S2, available as supplementary data at *Bioinformatics* online for details.

Cell Culture: Cell viability was assessed using the MCF7 and MCF10A cell lines. Cells were maintained in relevant media throughout the study, in an incubator set to 37°C and 5% CO_2_, with media changes every 2–3 days. Cells were passaged when reaching ∼80% confluency (typically every 3–4 days) using 1X TrypLE Select (MCF7) and PromoCell Detach Kit (MCF10A).

Compound plate preparation: Vehicle control (DMSO) or specified compounds was added to empty wells of a white-walled 384 well plate in a randomised fashion (one plate per cell line) using the D300e digital dispenser. Compound concentrations tested were 2.5 μM, 0.25 μM, 0.025 μM and 0.0025 μM. Well volumes were normalised to 404 nL with a consistent vehicle concentration of DMSO per well (0.5% (v/v) final assay DMSO concentration). Plates were sealed and stored at −4°C prior to use.

Viability Assay: Cells were seeded into compound-containing plates using the Dragonfly 10-Channel Dispenser at a density of 5.0 × 103 cells/cm^2^ (300 cells/well) in 40 μL of relevant growth medium. Plates were incubated for 48 h in a humidified incubator set to 37°C and 5% CO_2_. At the end of the incubation period, cells were equilibrated to 30°C and 40 μL of Cell Titer-Glo® 3D cell viability assay reagent was added (Promega; Cat #G9682). Plates were centrifuged briefly and incubated protected from light for 30 min at 30°C. Luminescence signal was measured using a CLARIOstar Plus microplate reader (BMG Labtech).

Data Analysis: Raw relative luminescence unit (RLU) data were de-randomised prior to background subtraction. Viability was determined as a percentage of vehicle control (DMSO only). % Viability = ((Sample RLU—Mean Positive Control) ÷ (Mean Negative Control—Mean Positive Control)) × 100.

## 3 Results

### 3.1 Drug response prediction and cell line selection

Predicting cell line responses to compounds plays an important role in drug discovery by providing insights into drug efficacy and mechanism of action. Omics-based approaches have been widely applied in this context, often relying on complex neural network architectures (Li *et al*. 2021, Tang *et al.* 2021, Liu and Mei 2023, Wang *et al*. 2024, Xia *et al*. 2024).

Here, we assessed the utility of DRPs (see Methods) as descriptors for predictive machine learning models (Fig. 1). Gradient boosting trees were selected for benchmarking based on their demonstrated efficacy in drug response modelling (Yang *et al*. 2019, Branson *et al*. 2024). Using the GDSC dataset, DRP-based descriptors were compared to PCA-transformed mRNA expression profiles. Both descriptor sets achieved strong predictive performance on the test set of 80 cell lines (Table 1). Notably, DRP descriptors consistently and significantly outperformed mRNA-based features across all metrics (paired t-test on RMSE, p < 0.001). Varying the size of the cell line panel (∼10, ∼30 and ∼100 cell lines; Table S3, available as supplementary data at *Bioinformatics* online), showed modest but significant gains in performance at each increment.

**Table 1:** Comparing performance between mRNA expression profiles and drug response profiles in predicting drug response for all drugs across 80 cell lines in the GDSC dataset.

Method	Pearson R	Spearman R	MSE	RMSE	MAE
DRP	0.904 ± 0.002	0.877 ± 0.002	0.246 ± 0.004	0.496 ± 0.004	0.370 ± 0.003
omics	0.834 ± 0.002	0.793 ± 0.004	0.434 ± 0.008	0.658 ± 0.006	0.503 ± 0.005

Models were trained using panels of ∼50 cell lines and ∼50 drugs; results are declared as averages along with standard deviations resulting from 5 independent experiments. Statistical significance of differences in performance between descriptor sets was assessed using a paired *t*-test on RMSE (*p* < 0.001).

Rather than constituting a strict leave-drug-out design, this framework minimises the exposure of the target drug during training by including it in only a small experimental panel, thereby reducing overestimation associated with conventional random splits that can include extensive measurements of the same compound. Even when restricting the panel to only 10 cell lines (Table S3, available as supplementary data at *Bioinformatics* online), DRP-based models outperformed previously published leave-drug-out approaches (Table S4, available as supplementary data at *Bioinformatics* online). Notably, models using omics features within the same limited-panel framework also exceeded the performance of these published methods, underscoring the value of focused, information-rich training panels.

We next evaluated the approach on the CCLE dataset, comprising 24 drugs tested across more than 500 cell lines. To enable comparison with previously published methods, 10-fold cross-validation was used. Within each fold, drugs were predicted individually. For each drug, 5% of training samples (∼25 cell lines) were randomly selected for training the models. The generated models were then tested on the held-out fold (comprising 10% of all cell lines). Panel selection was performed randomly rather than based on drug response due to the limited number of drugs in CCLE. This design reflects the intended application scenario: predicting drug responses across a large cell line collection after testing the compound in only a small experimental panel. Consistent with the GDSC results, DRP descriptors outperformed omics features across all performance metrics (Table 2).

**Table 2:** Experiments comparing DRP and omics features on the CCLE dataset using 10-fold cross validation with a limited cell line panel derived from the training folds.

Method	Pearson R	Spearman R	MSE	RMSE	MAE
DRP	0.861 ± 0.017	0.710 ± 0.024	0.198 ± 0.032	0.443 ± 0.035	0.247 ± 0.019
omics	0.784 ± 0.022	0.587 ± 0.013	0.302 ± 0.033	0.547 ± 0.031	0.328 ± 0.015

Statistical significance of differences in performance between descriptor sets was assessed using a paired *t*-test on RMSE (*p* < 0.001).

The analyses above report performance aggregated across all compounds. To assess variability at the individual drug level, we repeated the GDSC analysis using 10-fold cross-validation. Within each fold, a small subset of cell lines was selected from the training data using our panel selection method (Fig. 2). Performance was evaluated both globally (across all drug-cell line pairs) and per drug (Table 3). While overall performance remained strong, substantial variability was observed between individual compounds (Appendix A, available as supplementary data at *Bioinformatics* online). Notably, correlation metrics differed considerably between global and per-drug evaluation, whereas error metrics were more consistent, highlighting how they capture distinct aspects of model performance.

**Table 3:** Comparison of DRP-based model performance on the GDSC dataset using two evaluation approaches: overall (all data points combined) and per-drug (evaluating each drug individually).

Method	Pearson R	Spearman R	MSE	RMSE	MAE
overall	0.912 ± 0.002	0.887 ± 0.001	0.223 ± 0.004	0.472 ± 0.005	0.352 ± 0.003
per-drug	0.624 ± 0.149	0.612 ± 0.147	0.224 ± 0.042	0.455 ± 0.043	0.354 ± 0.033

Results are reported as averages ± standard deviations from 5 independent experiments.

To investigate factors influencing drug-level predictability, we focused on selective compounds, defined as those active in fewer than 25% of GDSC cell lines. Using a held-out set of 160 cell lines (∼20% of the dataset), we reformulated the task as binary classification (sensitivity defined as IC_50_ ≤ 1 µM). We identified 10 drugs whose models successfully recovered at least 75% of all positives, while requiring testing fewer than 30% of the 160 cell lines.

These high performing models corresponded to several kinase inhibitors with functionally related compounds in the training set, suggesting that shared target biology contributed to predictive performance (Table S5, available as supplementary data at *Bioinformatics* online). However, for drugs such as NG-25 (targeting TAK1 and MAP4K2), the top three compounds contributing to model performance had comparable feature importance scores but did not share annotated primary targets with NG-25. This result points to a more complex target interplay or potentially unknown shared mechanisms of action.

Extending this analysis to the 20 highest-performing models, we observed that in 10 cases the top-ranked predictive compound targeted a different molecule than the drug being predicted. In 8 of these cases, the top three most influential drugs all had different annotated targets from the predicted drug (Table S5, available as supplementary data at *Bioinformatics* online). Together, these observations indicate that DRP-based models can capture indirect biological relationships or polypharmacology, rather than relying solely on direct target similarity. This capability provides a mechanism-agnostic functional context for compounds under investigation, potentially facilitating the identification of unexpected biological connections.

### 3.2 DRP-guided cell line selection for biomarker discovery

Cell lines provide a controlled system for investigating molecular determinants of drug sensitivity. Although our study primarily used DRPs for efficient cell line selection, we explored whether the DRP-guided panels, along with the top and bottom predicted responders, could support transcriptome-based biomarker discovery, providing a compact framework for such analyses.

DRP-predicted sensitivities were used to construct an enriched cell line panel comprising the initially selected lines together with the top and bottom 10 predicted responders, resulting in ∼55–60 cell lines. This high-contrast subset was used to train random forest regression models based on mRNA expression for three high-performing drugs in GDSC: Refametinib and PD0325901 (MEK1/2 inhibitors), and QL-X-138 (BTK/MNK inhibitor). Key genes associated with drug response were identified using correlation-based feature selection, feature importance extraction, and SHAP analysis.

**Figure 3:**
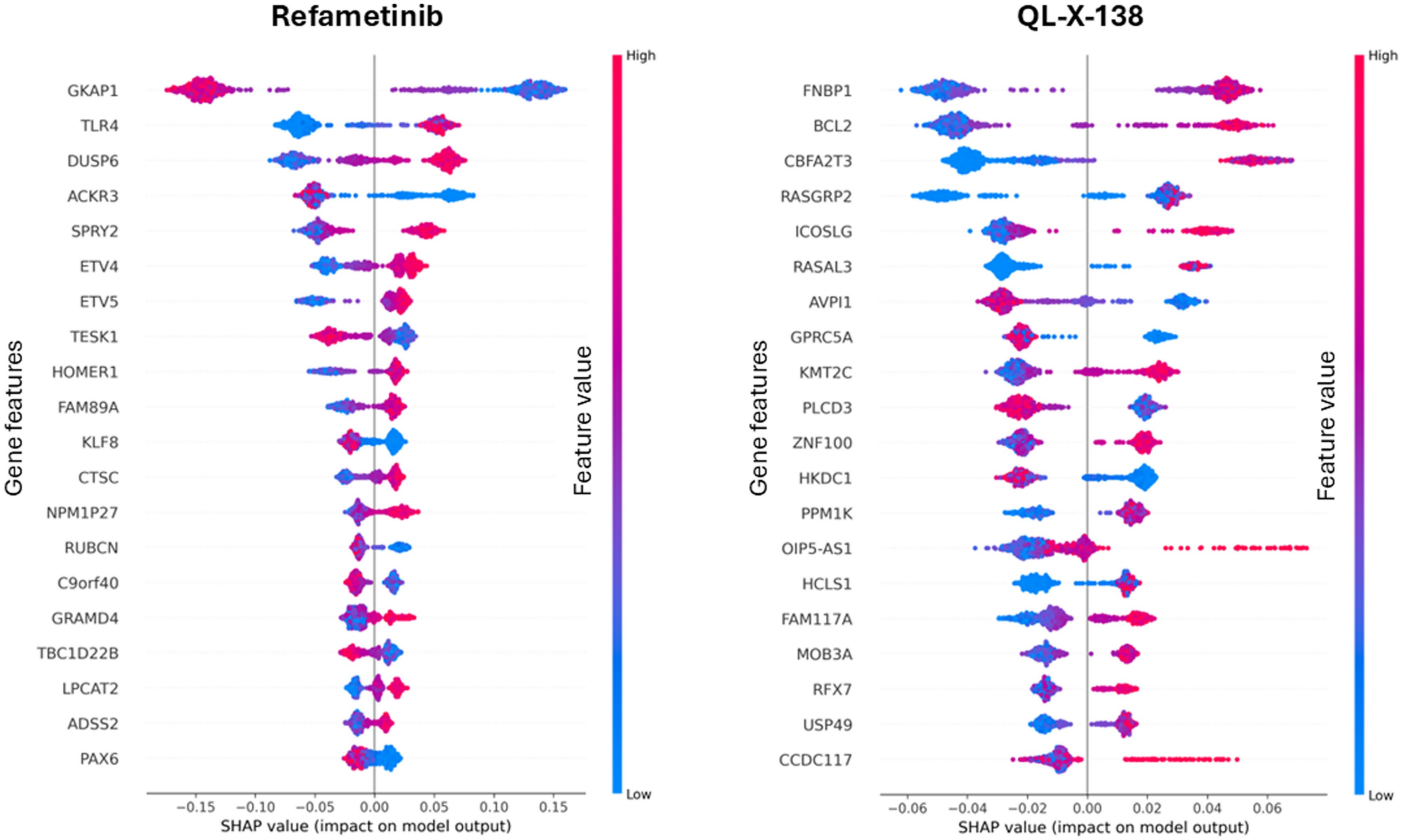
Uncovering biomarkers. SHAP bee swarm plot showing feature importances from a random forest model trained to predict cell line sensitivity to Refametinib and QL-X-138 using mRNA expression levels of the top 500 genes correlated with sensitivity. Results from the first of three replicate experiments are shown.

For Refametinib, expression of the direct targets (MEK1/2) was not predictive of sensitivity, consistent with previous reports. Instead, MAPK pathway regulators including ETV5, SPRY2, and TLR4 were among the most influential features (Fig. 3), in line with established MAPK-inhibitor sensitivity signatures and pathway rewiring mechanisms (Dry *et al*. 2010, Schubert *et al*. 2018). Excluding cell lines with low GKAP1 expression further enriched for sensitive lines, identifying GKAP1 as a potential biomarker with limited prior characterisation in cancer (Gallo *et al*. 2023). A comparable transcriptional signature was observed for PD0325901, supporting the consistency of the approach across related inhibitors.

For QL-X-138, the model exhibited greater variability across experiments. Nevertheless, it consistently highlighted markers such as WAS, RASAL3 and SEPTIN6 (positively associated with sensitivity) and PLCD3 and AVPI1 (negatively correlated) (Fig. 3). BCL2 frequently ranked among the top features consistent with reports linking BTK inhibition to increased BCL-2 dependence. The involvement of RASAL3 further suggests potential cross talk with the RAS/ERK pathway in response to BTK/MNK inhibition.

To assess whether compact DRP-derived panels capture broader biological signals, we compared feature importance profiles obtained from these focused panels with models trained on larger random subsets (70% of all cell lines). For MEK1/2 inhibitors Refametinib and PD0325901, there was substantial overlap in top SHAP-ranked genes (Fig. S1, available as supplementary data at *Bioinformatics* online), indicating that smaller, performance-driven panels retain key biological signatures. In contrast, QL-X-138 showed less feature overlap, consistent with its higher predictive variability and possibly amplified by highly correlated transcriptomic features.

### 3.3 Validating DRP versatility: compound prioritisation for selective toxicity

**Figure 4:**
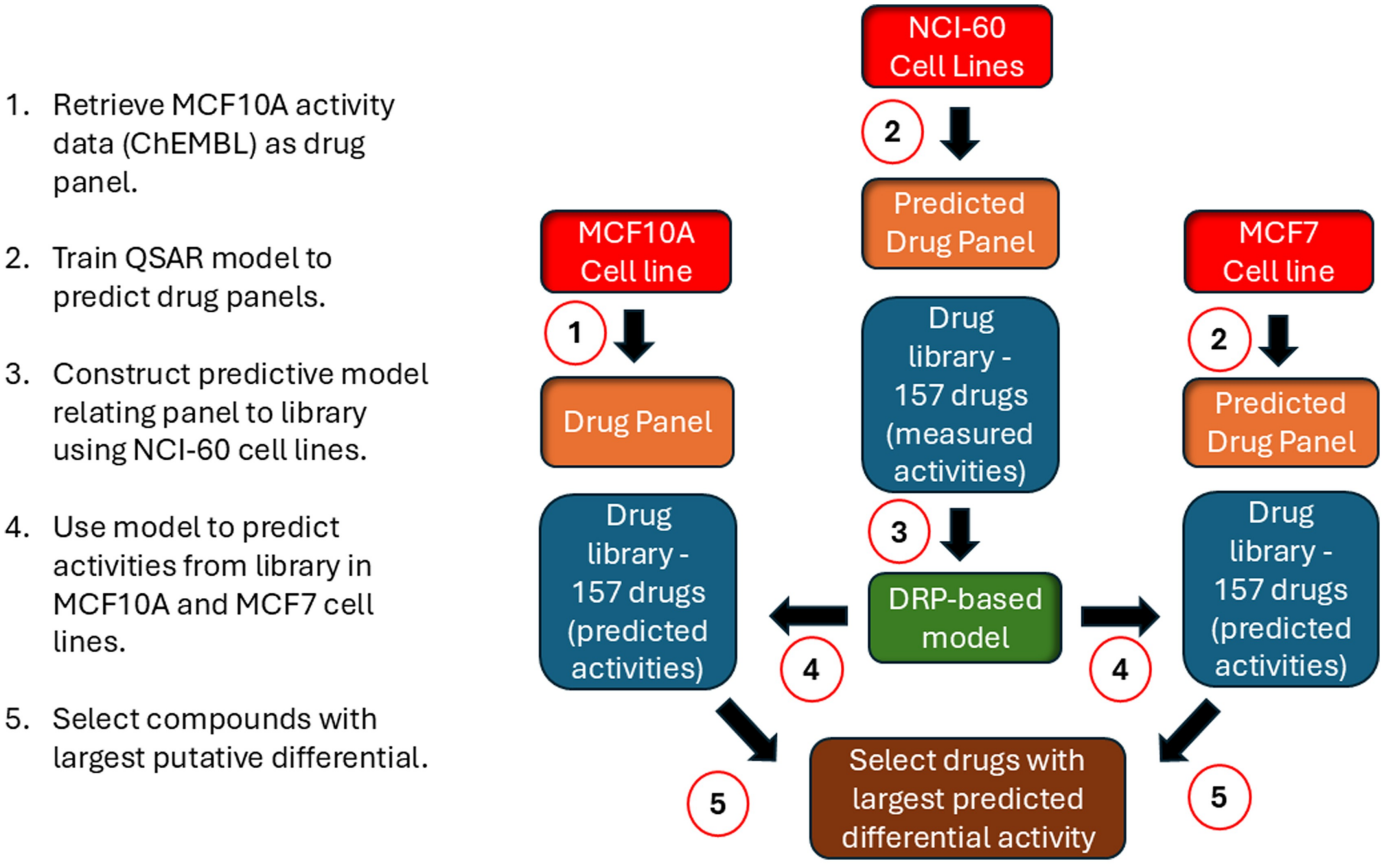
Workflow for generating putative MCF7 and MCF10A activity profiles from a 157-compound drug library. (1) MCF10A activity data were retrieved from ChEMBL to define a 223-compound drug panel. (2) QSAR models were trained to predict responses to these panel drugs across additional cell lines using NCI-60 data, generating a harmonised DRP feature space. (3) DRP-based models were trained using panel responses and measured library activity. (4) These models were used to predict responses to the 157-compound library for the target cell lines MCF10A and MCF7. (5) Compounds with the highest predicted differential activity, defined as max[(-log10(IC_50___MCF7_) – (-log10(IC_50___MCF10A_)], were selected for experimental validation.

Having demonstrated the utility of DRPs for cell line prioritisation and biomarker discovery, we next evaluated whether the framework could address a complementary objective: compound prioritisation for selective toxicity (Fig. 4). In contrast to predicting responses of many cell lines to a single drug, this experiment inverted the problem by predicting the responses of two specific cell lines (MCF7 and MCF10A) to a library of 157 compounds. The goal was to efficiently prioritise compounds that targeted the epithelial breast cancer cell line MCF7 while sparing the non-tumourigenic breast epithelial control line MCF10A, simulating an early-stage screening scenario in which therapeutic selectivity is key.

A methodological challenge was generating a consistent DRP feature set suitable for predicting MCF7 and MCF10A responses. Although ChEMBL provided activity data for a significant number of compounds tested on MCF10A, overlap with the broader cell line panel was limited. To harmonise the drug response profile across all cell lines, QSAR models based on MACCS fingerprints were trained to predict the activities of 223 MCF10A-measured compounds across all other cell lines. MCF10A retained experimentally measured values, while other cell lines were assigned QSAR-predicted responses, yielding a unified drug panel suitable for DRP construction.

Using this aligned drug panel, DRP-based models were trained to predict responses to the 157-compound library. Although experimental measurements were available for MCF7, predicted values were used for both cell lines to emulate a prospective early-screening scenario in which such data would be unavailable.

From the predicted responses, four compounds with the largest predicted differential effect (Δ viability favouring MCF7) were prioritised. We included two additional compounds predicted to show minimal or opposite toxicity as controls. These predicted differences were modest (less than tenfold), suggesting broadly similar responses between the two cell lines. All six compounds were tested experimentally across concentrations ranging from 2.5 μM down to 2.5 nM.

The experimental results successfully identified two selective agents, Pevonedistat and BI 2536, which were among the four prioritised compounds (Table 4). Both achieved IC_50_ values below 100 nM in MCF7. In the control line MCF10A, BI 2536 had an IC_50_ closer to 1 μM and Pevonedistat exceeded 2.5 μM. The two negative controls (Pemetrexed and AZD6482) did not reach IC_50_ values in either cell line, consistent with predictions, although MCF10A was slightly more sensitive to AZD6482 at the highest concentration tested.

**Table 4:** Differential drug response predictions in MCF7 and MCF10A.

Drug	Predicted Preference Δ(−log10(IC_50_))	Measured Preference	MCF7 IC_50_ Reached	MCF10 IC_50_ Reached
**Pevonedistat**	MCF7 (>2)	MCF7	Y	N
Gemcitabine	MCF7 (>2)	MCF10	N	Y
Cytarabine	MCF7 (>2)	None	N	N
**BI 2536**	MCF7 (>2)	MCF7	Y	Y
**Pemetrexed**	None (<1)	None	N	N
**AZD 6482**	None (<1)	None	N	N

Predicted and measured sensitivities for six tested drugs are shown, including predicted differential activity (Δ(-log10(IC_50_))), experimental outcomes (*n* =3)), and whether IC_50_ was reached at ≤2.5 μM. Drugs shown in bold indicate predictions that were consistent with experimental results.

Some misclassifications were observed. Gemcitabine, predicted to favour MCF7, showed higher potency in MCF10A (IC_50_ < 0.25 μM), while Cytarabine displayed minimal differential activity, with both cell lines maintaining over 70% viability at 2.5 μM. Complete viability data are provided in Table S6, available as supplementary data at *Bioinformatics* online.

## 4 Discussion

Our study demonstrates that drug response profiles (DRP) descriptors can be used with machine learning to predict cell line sensitivity across diverse compounds. Using multiple datasets and benchmarking, we demonstrated the practical utility and generalisability of DRP-based features. The value of the DRP approach lies in the type of information it encodes. Unlike static omics features, which provide a molecular snapshot, DRPs are derived from pharmacological perturbation responses and therefore reflect the integrated effects of pathway activity, feedback regulation, and compensatory adaptation. In this sense, DRPs provide a functional systems-level readout of drug response that complements molecular profiling and captures aspects of cellular behaviour that are not directly observable from static features alone. This functional encoding enables predictive modelling in comparatively low-data settings. Practically, new cell lines can be profiled against a compact compound panel to infer broader sensitivities, and novel drugs can be evaluated in a small, information-rich cell line subset to estimate their wider activity landscape.

We further show that DRP-guided cell line selection can support mechanistic interpretation when paired with omics-based models and explainability techniques like SHAP. For MEK1/2 inhibitors, established MAPK pathway regulators (including ETV5 and SPRY2) were recovered, consistent with known feedback and resistance mechanisms, and GKAP1 emerged as a potential additional biomarker candidate. For the BTK/MNK dual inhibitor, recurrent identification of BCL2 and RASAL3 suggest pathway dependencies and possible RAS/ERK cross talk. Notably, these signals were detectable using compact, performance-driven panels, indicating that biologically meaningful insights can be retrieved even with reduced experimental scope.

We also extended the framework to compound prioritisation for selective toxicity. In a prospective setting, DRP-based models identified Pevonedistat and BI 2536 as selective agents for MCF7 relative to MCF10A, while correctly deprioritising negative controls. Although misclassifications were observed, the results illustrate that DRP features can support early-stage compound triage when experimental screening resources are limited.

At the same time, performance variability across drugs highlights important methodological considerations. Differences in RMSE suggest that predictive accuracy depends on the informativeness and composition of both the drug and cell line panels. Future work should therefore focus on systematically optimising panel design and evaluating performance across broader and more heterogeneous datasets.

Overall, these findings position DRP-based modelling as a functional complement to omics approaches, offering a robust and cost-effective platform to accelerate mechanism-informed drug discovery.

## 5 Conclusion

This study introduces a validated, practical framework for accelerating early-stage drug development through Drug Response Profile (DRP) descriptors. By providing an orthogonal, functionally agnostic feature set, the DRP approach can support critical decisions in discovery pipelines, ranging from mechanistic biomarker discovery to the efficient prioritisation of selective drug candidates. Ultimately, this framework offers a cost-effective and scalable strategy for early-stage compound prioritisation in targeted therapy development.

## Supplementary Material

btag293_Supplementary_Data

## Data Availability

Data uploaded to https://github.com/abbiAR/-Strategic-Cell-Line-and-Compound-Selection-Using-Drug-Response-Profiles

## References

[btag293-B1] Abdel-Rehim A , Orhobor O, Griffiths G et al Establishing predictive machine learning models for drug responses in patient derived cell culture. NPJ Precis Oncol 2025;9:180. 40514399 10.1038/s41698-025-00937-2 PMC12166088

[btag293-B2] Adashek JJ , Kato S, Sicklick JK et al If it’s a target, it’s a pan-cancer target: tissue is not the issue. Cancer Treat Rev 2024;125:102721. 38522181 10.1016/j.ctrv.2024.102721 PMC11093268

[btag293-B3] Baião AR , Cai Z, Poulos RC et al A technical review of multi-omics data integration methods: from classical statistical to deep generative approaches. Brief Bioinform 2025;26:bbaf355. 40748323 10.1093/bib/bbaf355 PMC12315550

[btag293-B4] Barretina J , Caponigro G, Stransky N et al The cancer cell line encyclopedia enables predictive modelling of anticancer drug sensitivity. Nature 2012;483:603–7. 22460905 10.1038/nature11003 PMC3320027

[btag293-B5] Branson N , Cutillas PR, Bessant C et al Comparison of multiple modalities for drug response prediction with learning curves using neural networks and XGBoost. Bioinform Adv 2024;4:vbad190. 38282976 10.1093/bioadv/vbad190 PMC10812874

[btag293-B6] Dolgin E. The future of precision cancer therapy might be to try everything. Nature 2024;626:470–3. 38356072 10.1038/d41586-024-00392-2

[btag293-B7] Dry JR , Pavey S, Pratilas CA et al Transcriptional pathway signatures predict MEK addiction and response to selumetinib (AZD6244). Cancer Res 2010;70:2264–73. 20215513 10.1158/0008-5472.CAN-09-1577 PMC3166660

[btag293-B8] Gallo S , Vitacolonna A, Crepaldi T et al NMDA receptor and its emerging role in cancer. Int J Mol Sci 2023;24:2540. 36768862 10.3390/ijms24032540 PMC9917092

[btag293-B9] Gonçalves E , Segura-Cabrera A, Pacini C et al Drug mechanism-of-action discovery through the integration of pharmacological and CRISPR screens. Mol Syst Biol 2020;16:e9405. 32627965 10.15252/msb.20199405 PMC7336273

[btag293-B10] Goodspeed A , Heiser LM, Gray JW et al Tumor-derived cell lines as molecular models of cancer pharmacogenomics. Mol Cancer Res 2016;14:3–13. 26248648 10.1158/1541-7786.MCR-15-0189 PMC4828339

[btag293-B11] Gouda MA , Nelson BE, Buschhorn L et al Tumor-agnostic precision medicine from the AACR GENIE database: clinical implications. Clin Cancer Res 2023;29:2753–60. 37061987 10.1158/1078-0432.CCR-23-0090 PMC10390861

[btag293-B12] Iorio F , Knijnenburg TA, Vis DJ et al A landscape of pharmacogenomic interactions in cancer. Cell 2016;166:740–54. 27397505 10.1016/j.cell.2016.06.017 PMC4967469

[btag293-B13] Jordan EJ , Kim HR, Arcila ME et al Prospective comprehensive molecular characterization of lung adenocarcinomas for efficient patient matching to approved and emerging therapies. Cancer Discov 2017;7:596–609. 28336552 10.1158/2159-8290.CD-16-1337 PMC5482929

[btag293-B14] Li M , Wang Y, Zheng R et al DeepDSC: a deep learning method to predict drug sensitivity of cancer cell lines. IEEE/ACM Trans Comput Biol Bioinform 2021;18:575–82. 31150344 10.1109/TCBB.2019.2919581

[btag293-B15] Li Y , Umbach DM, Krahn JM et al Predicting tumor response to drugs based on gene-expression biomarkers of sensitivity learned from cancer cell lines. BMC Genomics 2021;22:272. 33858332 10.1186/s12864-021-07581-7 PMC8048084

[btag293-B16] Liu X-Y , Mei X-Y. Prediction of drug sensitivity based on multi-omics data using deep learning and similarity network fusion approaches. Front Bioeng Biotechnol 2023;11:1156372. 37139048 10.3389/fbioe.2023.1156372 PMC10150883

[btag293-B17] Menden MP , Wang D, Mason MJ et al Community assessment to advance computational prediction of cancer drug combinations in a pharmacogenomic screen. Nat Commun 2019;10:2674. 31209238 10.1038/s41467-019-09799-2 PMC6572829

[btag293-B18] Moshkov N , Becker T, Yang K et al Predicting compound activity from phenotypic profiles and chemical structures. Nat Commun 2023;14:1967. 37031208 10.1038/s41467-023-37570-1 PMC10082762

[btag293-B19] Pedregosa F , Varoquaux G, Gramfort A et al Scikit-learn: machine learning in python. J Mach Learn Res 2011;12:2825.

[btag293-B20] Schubert M , Klinger B, Klünemann M et al Perturbation-response genes reveal signaling footprints in cancer gene expression. Nat Commun 2018;9:20. 29295995 10.1038/s41467-017-02391-6 PMC5750219

[btag293-B21] Tang YC , Gottlieb A. Explainable drug sensitivity prediction through cancer pathway enrichment. Sci Rep 2021;11:3128. 33542382 10.1038/s41598-021-82612-7 PMC7862690

[btag293-B22] Wahida A , Buschhorn L, Fröhling S et al The coming decade in precision oncology: six riddles. Nat Rev Cancer 2023;23:43–54. 36434139 10.1038/s41568-022-00529-3

[btag293-B23] Wang Y , Yu X, Gu Y et al XGraphCDS: an explainable deep learning model for predicting drug sensitivity from gene pathways and chemical structures. Comput Biol Med 2024;168:107746. 38039896 10.1016/j.compbiomed.2023.107746

[btag293-B24] Xia X , Zhu C, Zhong F et al TransCDR: a deep learning model for enhancing the generalizability of drug activity prediction through transfer learning and multimodal data fusion. BMC Biol 2024;22:227. 39385185 10.1186/s12915-024-02023-8 PMC11462810

[btag293-B25] Yang M , Tao B, Chen C et al Machine learning models based on molecular fingerprints and an extreme gradient boosting method lead to the discovery of JAK2 inhibitors. J Chem Inf Model 2019;59:5002–12. 31746601 10.1021/acs.jcim.9b00798

