# Supplementary material for: Drug response profile-based machine learning enables strategic cell line and compound selection for drug development
Source: Bioinformatics. 2026 May 8;42(6):btag293. doi: 10.1093/bioinformatics/btag293 (PMC13242297; doi:10.1093/bioinformatics/btag293)
Supplement: btag293_Supplementary_Data [file btag293_supplementary_data.zip › Supplementary_info.pdf]

Supplementary Information

Advancing Drug Development: Predicting Cancer Cell Line Sensitivity with Drug Response Panels and Machine Learning

Descriptor	*depth*	*trees*	*PEARSON*	*SPEARMAN*	*RMSE*	*MSE*	*MAE*
DRP	2	100	0.938±0.002	0.892±0.004	0.397±0.006	0.158±0.005	0.302±0.004
DRP	2	50	0.939±0.002	0.892±0.003	0.395±0.005	0.157±0.004	0.301±0.004
DRP	3	100	0.937±0.001	0.891±0.004	0.399±0.005	0.159±0.004	0.303±0.003
DRP	3	50	0.938±0.002	0.892±0.004	0.397±0.006	0.158±0.005	0.302±0.004
DRP	4	100	0.936±0.002	0.889±0.003	0.402±0.006	0.162±0.005	0.305±0.003
DRP	4	50	0.937±0.002	0.89±0.003	0.402±0.006	0.161±0.005	0.305±0.004
OMICS	2	100	0.868±0.004	0.772±0.006	0.573±0.009	0.328±0.01	0.442±0.004
OMICS	2	50	0.871±0.004	0.776±0.006	0.568±0.008	0.322±0.009	0.438±0.004
OMICS	3	100	0.869±0.005	0.773±0.007	0.572±0.01	0.328±0.011	0.44±0.007
OMICS	3	50	0.87±0.004	0.775±0.006	0.57±0.008	0.325±0.01	0.439±0.006
OMICS	4	100	0.869±0.005	0.775±0.009	0.571±0.009	0.326±0.01	0.439±0.008
OMICS	4	50	0.869±0.005	0.775±0.009	0.571±0.009	0.326±0.01	0.439±0.007

Table S1. Evaluation of impact on performance for the two descriptor sets (DRP, OMICS) using different parameter settings for the gradient boosting tree learner. A dedicated validation set of 80 patients was used.

Trametinib	Pazopanib	Oxyphenisatin acetate	Selumetinib	Podofilox
Brilanestrant	BMS-345541	AMG319	MIM1	Clofarabine
Regorafenib	BAY885	Mitoxantrone	WZ4003	Ruxolitinib
AZ6102	Obatoclax (Mesylate)	VX-11e	Paclitaxel	Apabetalone
Tamoxifen	AZD4573	YK-4-279	Epothilone B	Ipatasertib
ZM-447439	ULK-101	Lomustine	Gemcitabine	K-Ras(G12C) inhibitor 12
Foretinib	Linsitinib	AZD5582	Sabutoclax	Afuresertib
(+)-JQ-1	Everolimus	Vps34-IN-1	LGK974	BIBR 1532
AZD4547	Cytarabine	P 22077	Ceralasertib	LCL161
BMS-754807	Rapamycin	Etoposide	Birabresib	Leflunomide
OSI-027	Irinotecan	Tozasertib	Pevonedistat	Uprosertib
Fulvestrant	SB-505124	BMS-536924	AGI-6780	UMI-77
Sepantronium (bromide)	Carmustine	Flavopiridol	LJI308	Dabrafenib
Sapitinib	Molibresib	GSK591	Solcitinib	MN-64
NVP-ADW742	Vandetanib	Entinostat	ABT-737	SCH772984
Doramapimod	Alisertib	AZD-5991	CZC24832	PF-4708671
Bosutinib	Ponatinib	Teniposide	Ganetespib	I-BRD9
Taselisib	LY2109761	Dinaciclib	GSK2606414	WIKI4
AZD-7762	GSK2578215A	KU-57788	Vistusertib	Crizotinib
Vorinostat	Vemurafenib	Olaparib	Fludarabine	MST-312
Dasatinib	AZD 6482	Nintedanib	Osimertinib	LCL161

Afatinib	KU-55933	EPZ004777	Temozolomide	Lapatinib
AZD-8055	GSK343	VE-821	Erlotinib	GSK269962A
Sorafenib	Luminespib	XAV-939	Docetaxel	AGI-5198
OF-1	AZD-5438	Ulixertinib	BI 2536	(Z)-Mirin
Nutlin-3a	Palbociclib	Savolitinib	FRAX486	AZD1208
Nelarabine	Ibrutinib	Zorifertinib	Ravoxertinib	5-Fluorouracil
Cediranib	Cladribine	Talazoparib	Dihydrorotenone	Staurosporine
Bortezomib	AZ960	ML-323	PCI-34051	IWP-2
PD173074	AZD8186	Wnt-C59	Pictilisib	Oxaliplatin
AZD-1480	Adavosertib	Camptothecin	Buparlisib	Brigatinib
BAY-299	Navitoclax	Pinometostat	Gefitinib	Carfilzomib
GNE-317	Vismodegib	Ribociclib	Neratinib	MG-132
Nilotinib	SB 216763	AT13148	PF-06650833	Carboplatin
Pemetrexed	GSK1904529A	Capivasertib	PLX-4720	Cisplatin
Mirdametinib	PFI-3	PCL 016	Entospletinib	Cyclophosphamide
Niraparib	Ro-3306	Eprenetapopt	SCH900776	Dactolisib
Axitinib	Sunitinib	Alpelisib	Venetoclax	

Table S2. Drug library used for drug screening experiments.

Descriptor	*cell line panel size*	*PEARSON*	*SPEARMAN*	*MSE*	*RMSE*	*MAE*
DRP	10	0.848±0.008	0.813±0.007	0.386±0.021	0.621±0.017	0.465±0.013
OMICS	10	0.801±0.005	0.755±0.007	0.509±0.018	0.714±0.012	0.546±0.009
DRP	30	0.892±0.002	0.864±0.002	0.276±0.006	0.525±0.006	0.392±0.006
OMICS	30	0.811±0.006	0.766±0.008	0.490±0.013	0.700±0.009	0.533±0.008
DRP	50	0.904±0.002	0.877±0.002	0.246±0.004	0.496±0.004	0.370±0.003
OMICS	50	0.834±0.002	0.793±0.004	0.434±0.008	0.658±0.006	0.503±0.005
DRP	100	0.921±0.001	0.897±0.001	0.204±0.001	0.452±0.001	0.334±0.001
OMICS	100	0.857±0.001	0.819±0.001	0.362±0.003	0.602±0.003	0.456±0.003

Table S3. Comparing performance for the two descriptor sets (DRP, OMICS) on the GDSC dataset using different cell line panel sizes and a dedicated test-set of 80 patients.

Method	$\text{RMSE}_{\log10(\text{IC50})}$	$\text{RMSE}_{\ln(\text{IC50})}$
DRPi (10)	0.621	1.438
PathDSP	0.98	-
DeepDSC	1.24	-
XGraphCDS	-	1.726

Table S4. Comparison of the drug response panel inference (DRPi) method, using 10 cell lines in the panel, against three other published studies (leave-drug-out scenarios) [1, 2, 3].

Compound	Primary target(s)	sum top 3 feature importances	top 3 important features from drug panel	fractional share of feature importance (total=1)	primary targets of features
Osimertinib	EGFR	0.612	Sapitinib	0.384	EGFR, ERBB2, ERBB3
			Entinostat	0.12	HDAC1, HDAC3
			P22077	0.108	USP7, USP47
Afatinib	EGFR, ERBB2	0.823	Sapitinib	0.58	EGFR, ERBB2, ERBB3
			Pelitinib	0.134	EGFR
			Amuvatinib	0.109	KIT, PDGFRA, FLT3
Refametinib	MEK1, MEK2	0.82	PD0325901	0.726	MEK1, MEK2
			(5Z)-7-Oxozeaenol	0.049	TAK1
			PRT062607	0.045	SYK
ABT737	BCL2, BCL-XL, BCL-W, BCL-B, BFL1	0.919	Navitoclax	0.429	BCL2, BCL-XL, BCL-W
			Venetoclax	0.393	BCL2
			WEHI-539	0.096	BCL-XL
**JW-7-24-1**	LCK	0.768	QL-X-138	0.619	BTK
			Masitinib	0.089	KIT, PDGFRA, PDGFRB
			Enzastaurin	0.061	PKCB
Venetoclax	BCL2	0.742	Navitoclax	0.462	BCL2, BCL-XL, BCL-W
			PCI-34051	0.208	HDAC8, HDAC6, HDAC1
			Y-39983	0.072	ROCK
PD0325901	MEK1, MEK2	0.891	Refametinib	0.676	MEK1, MEK2
			Ulixertinib	0.176	ERK1, ERK2
			CI-1040	0.038	MEK1, MEK2
Navitoclax	BCL2, BCL-XL, BCL-W	0.864	ABT737	0.781	BCL2, BCL-XL, BCL-W, BCL-B, BFL1
			WEHI-539	0.067	BCL-XL
			Sapitinib	0.016	EGFR, ERBB2, ERBB3
Taselisib	PI3K (beta sparing)	0.789	Alpelisib	0.681	PI3Kalpha
			OSI-027	0.063	MTORC1, MTORC2
			Ipatasertib	0.045	AKT1, AKT, AKT3
**NG-25**	TAK1, M4K2	0.604	CX-5461	0.24	RNA Polymerase 1
			YM201636	0.193	PIKFYVE
			Nilotinib	0.172	ABL
JQ1	BRD2, BRD3, BRD4, BRDT	0.661	OTX015	0.515	BRD2, BRD3, BRD4
			Entinostat	0.078	HDAC1, HDAC3

			Foretinib	0.068	MET, KDR, TIE2, VEGFR3/FLT4, RON,
Vorinostat	HDAC inhibitor Class I, IIa, IIb, IV	0.556	PRT062607	0.229	SYK
			PCI-34051	0.215	HDAC8, HDAC6, HDAC1
			Erlotinib	0.112	EGFR
SCH772984	ERK1, ERK2	0.706	PD0325901	0.564	MEK1, MEK2
			Ulixertinib	0.093	ERK1, ERK2
			JQ12	0.049	HDAC1, HDAC2
**Alisertib**	AURKA	0.85	AZ960	0.755	JAK2, JAK3
			AZD6738	0.054	ATR
			Pyridostatin	0.041	G-quadruplex stabiliser
**Bosutinib**	SRC, ABL, TEC	0.5	PRT062607	0.28	SYK
			Sapitinib	0.141	EGFR, ERBB2, ERBB3
			WZ4003	0.079	NUAK1, NUAK2
**QL-X-138**	BTK	0.875	JW-7-24-1	0.654	LCK
			GSK690693	0.2	AKT1, AKT2, AKT3
			WZ4003	0.021	NUAK1, NUAK2
**PHA-793887**	CDK2, CDK7, CDK5	0.685	Masitinib	0.372	KIT, PDGFRA, PDGFRB
			YM201636	0.2	PIKFYVE
			QL-X-138	0.113	BTK
**Irinotecan**	TOP1	0.842	Talazoparib	0.748	PARP1, PARP2
			Foretinib	0.048	MET, KDR, TIE2, VEGFR3/FLT4, RON, PDGFR, FGFR1, EGFR
			AZD6738	0.046	ATR
Pelitinib	EGFR	0.557	Tipifarnib	0.314	Farnesyl-transferase (FNTA)
			Sapitinib	0.18	EGFR, ERBB2, ERBB3
			WEHI-539	0.064	BCL-XL
**Talazoparib**	PARP1, PARP2	0.852	Irinotecan	0.669	TOP1
			SB52334	0.099	ALK5
			Entinostat	0.083	HDAC1, HDAC3

Table S5. Top three compound features for 20 response prediction models. The table display the drug that is being predicted, the main declared targets of the drug, the fraction of the combined top 3 features (based on sci-kit learn random forest feature importance). The top three features (compounds) based on importance scores. The primary targets of each featured compound.

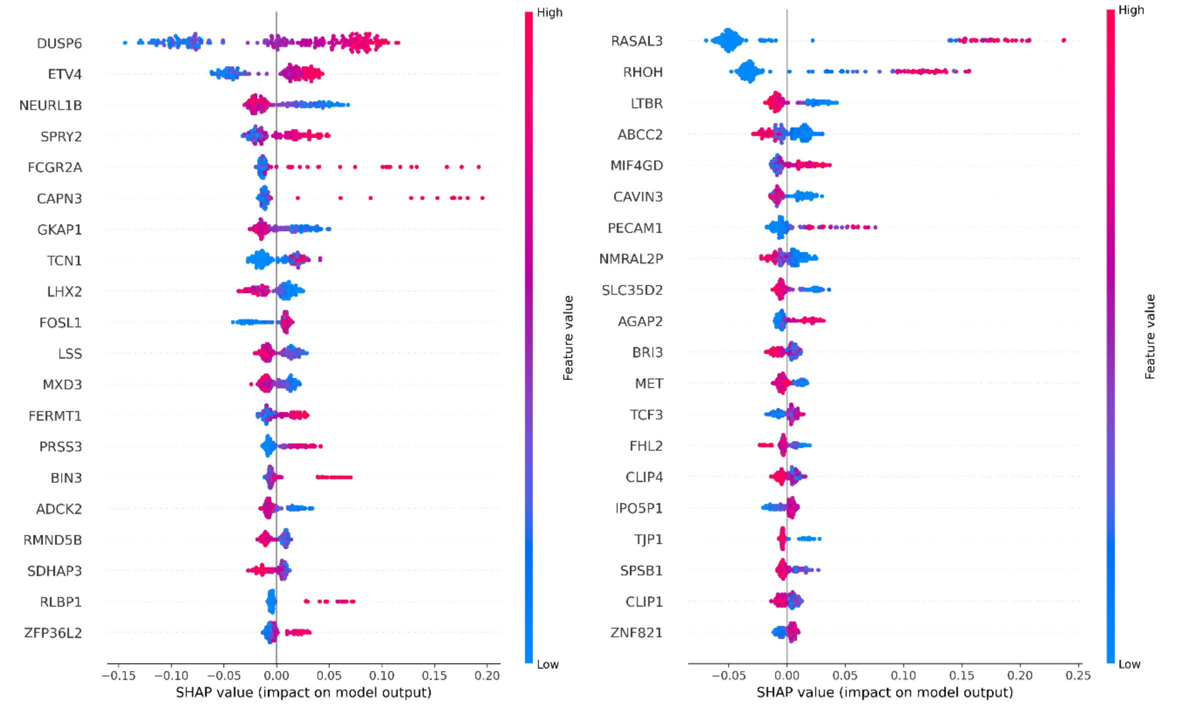

Figure S1. SHAP bee swarm plot showing feature importances from a random forest model trained to predict cell line sensitivity to Refametinib and QL-X-138 using mRNA expression levels of the top 500 genes correlated with sensitivity. This experiment utilised 70% of the data in training. Results from the first of three replicate experiments are shown.

Drug	predicted pXIC50 differential	cell line	2.5 uM	0.25 uM	0.025 uM	0.0025 uM
**Cytarabine**	2.41	MCF7	75.28	88.39	106.86	94.11
		MCF10A	76.86	77.41	90.31	92.85
**Gemcitabine**	2.21	MCF7	79.94	71.58	70.27	83.25
		MCF10A	45.52	79.25	80.2	87.49
**BI 2536**	2.49	MCF7	29.69	44.58	59.07	68.48
		MCF10A	33.85	72.27	67.67	92.4
**Pevonedistat**	2.04	MCF7	22.05	38.68	58.81	81.02
		MCF10A	60.61	63.92	69.74	95.55
**Pemetrexed**	-0.84	MCF7	57.92	69.2	82.72	99.78
		MCF10A	57.6	79.69	97.15	107.26
**AZD 6482**	-0.69	MCF7	71.43	104.84	98.02	107.43
		MCF10A	83.56	96.19	105.66	108.21

Table S6. Drug responses from experimental validation results of single agents with predicted differential effects on the tumorigenic breast cancer cell line MCF7 and the non-tumorigenic MCF10A. Viability values are shown for both cell lines. Control compounds Pemetrexed and AZD6482 were predicted to show no differential effect.

Supplementary Information references

1. Tang, Y. C., & Gottlieb, A. (2021). Explainable drug sensitivity prediction through cancer pathway enrichment. *Scientific reports*, *11*(1), 3128. https://doi.org/10.1038/s41598-021-82612-7

2. Li, M., Wang, Y., Zheng, R., Shi, X., Li, Y., Wu, F. X., & Wang, J. (2021). DeepDSC: A Deep Learning Method to Predict Drug Sensitivity of Cancer Cell Lines. *IEEE/ACM transactions on computational biology and bioinformatics*, *18*(2), 575–582. https://doi.org/10.1109/TCBB.2019.2919581

3. Wang, Y., Yu, X., Gu, Y., Li, W., Zhu, K., Chen, L., Tang, Y., & Liu, G. (2024). XGraphCDS: An explainable deep learning model for predicting drug sensitivity from gene pathways and chemical structures. *Computers in biology and medicine*, *168*, 107746. https://doi.org/10.1016/j.compbiomed.2023.107746
